# Supplementary material for: The impact of insurance institutional investors on corporate value from selection and creation perspective
Source: PLoS One. 2022 Jul 1;17(7):e0269520. doi: 10.1371/journal.pone.0269520 (PMC9249200; doi:10.1371/journal.pone.0269520)
Supplement: Attachment — Submitted filename: Responses to Reviewer 2022_03_23 (final).docx [file pone.0269520.s002.docx]

# Responses to Reviewer1:

**The Impact of Insurance Institutional Investors on Corporate Value from Selection and Creation Perspective**

Our sincere gratitude to the reviewer for the constructive comments on our manuscript. Your constructive comments on the paper were extremely valuable in improving the quality of the paper. We have carefully revised the manuscript and adopted all of your suggestions. In what follows, your comments are shown in italics and followed by our point-by-point responses. All the page numbers refer to those in the revision. Changes made according to the Major Comments in the revised manuscript are highlighted in blue color of the revised paper for ease of your reference. We have made numerous minor changes throughout the paper, according to your special comments. We believe that you will find this report and our revised manuscript have satisfactorily addressed the issues you have raised and that the revised manuscript meets the high standards of PLOS One. We thank you in advance for your valuable time in reviewing the manuscript.

**Reviewer’s Comment (Major Comment 1):**

*1. Abstract*

*I suggest author(s) make an extra effort to write a more attractive abstract. It would be interesting to at least state the main objective of the study and highlight the most important findings. For example, you state that ‘The impact that insurance institutional investors have on corporations is an urgent problem addressed in academic and practical research’, but why it is an urgent problem? In my opinion, this is not clear in the summary, nor in the full article.*

**Authors’ Response (Major Comment 1):**

Thank you for the reviewer's valuable comments. We entirely rewrote the abstract in response to the recommendation. The main objective, the most important findings, and the importance of exploring the issue are all highlighted in the revised manuscript. The reason we believe this is a pressing issue is that, following the "Vanke-Baoneng Battle" in China, society views insurance funds as "barbaric funds", making enterprises undesirable and even fearful of insurance institutional investors. However, there are few distinct evaluations of insurance institutional investors, which are all combined with the entire institutional investors, both theoretically and empirically. This section has now been included to the introduction, and the "Vanke-Baoneng Battle" incident has been explained. The following changes have been made to the abstract, which are highlighted in blue in the revised manuscript:

Abstract: The majority of insurance investment funds are derived from policy liability debt funds. It differs from other institutional investors in a number of ways, including investment size, horizon, duration, risk, and so on. However, only a small portion of the extant literature focuses on in-depth and extensive analysis of Insurance Institutional Investors' holdings (IIIs). This study analyses the impact of shareholding by insurance institutions on the value of Shanghai and Shenzhen A-share listed companies in China's capital market. The paper offers three major contributions. First, we discovered that long-term equity-holding IIIs have both value selection and value creation functions. Second, the value creation function becomes more significant among long-term stock-holding IIIs with an increase in the period during which they retain the company's shares; Third, fast-in and fast-out (FIFO) IIIs have a value-inhibiting effect on the held company and serve a value selection role, rather than a value creation function. This study rectifies the lack of academic interest in insurance institutions and serves as a foundation and reference for the design of regulatory policies for insurance institutions' involvement in stock markets. It also gives empirical evidence for corporations to accurately analyze shareholding by insurance institutions. Furthermore, since this study concentrates on China's capital market, it can serve as a benchmark for other nations, particularly, those designated as developing market economies.

***Reviewer’s Comment (Major Comment 2):***

*2. Introduction*

***Reviewer’s Comment (Major Comment 2-(1)):***

*The introduction is extremely poor. It is not clearly explained why this study is necessary. The goals pursued are also unclear. Until the ‘Research Hypothesis’ section, it did not seem very clear to me what specific goals the author(s) are addressing in this article. I encourage author(s) to explicitly state the research goals/questions and develop the motivation for why it is necessary to carry them out. To this end, I suggest the following:*

*（1） Rewrite the introduction following the traditional structure of 2 pages and 4 paragraphs: (1) review of what we know about the research topic; (2) identification of research gaps that your research fills; (3) research objectives and brief description of how author intends to achieve those objectives; and (4) contributions to the literature.*

**Authors’ Response (Major Comment 2-(1)):**

We are grateful for such specific and detailed suggestions. We rearranged and rewrote the introduction in response to the reviewer's suggestions. The present introduction is structured as follows, with clear writing for each component suggested by reviewers, and the updated introduction is shown in the blue in the revised manuscript.

The first paragraph discusses the significance and peculiarity of insurance funds, as well as the issues that arise from their holdings in practise. This is our motivation for writing this article.

The second paragraph summarizes our knowledge of the research issue. It is noted that there are very few research on the heterogeneity of institutional investors in the available literature, with a clear lack of attention paid to insurance institutional investors.

The third paragraph lists the research gaps that our study addresses.

The fourth paragraph contains research objectives as well as a brief summary of how we plan to attain them. There's also a reason why we chose to conduct research on China's capital market or insurance institutional investors.

The fifth paragraph introduces the article's research findings and contributions to the literature.

***Reviewer’s Comment (Major Comment 2-(2)):***

*（2）Include calls from scholars for research on this topic and explain why these scholars have called for research on this topic. The paper focuses on China, but why China? Justifying the context in which your study takes place, as well as explaining why this context is appropriate for analysing your research question, can improve the quality of the manuscript and help readers better understand the results.*

**Authors’ Response (Major Comment 2-(2)):**

We appreciate for your comment on raising this very important issue. As the reviewer stated, fully stating the history of the research and why this subject was examined in China will assist improve the paper's quality and help readers better grasp the paper's results. As a result, in the introduction, we augment the research background and why we examine China as follows (last paragraph on p. 5):

The study location was chosen as China for two reasons: First, China has the world's second-largest insurance market, behind only the United States. Simultaneously, China's insurance market has been ranked first in terms of growth rate among the top 20 insurance markets in the world from 2011 to 2020. Additionally, Chinese insurance funds' investments in A-shares accounted for 3.44 percent of the total market value at the end of the third quarter of 2019, and their bond investments accounted for 6.49 percent of the total size of China's bond market, making them the second largest institutional investor in the Chinese market after public funds. Second, as a growing economy, compared with the developed countries, China's insurance industry started later but experienced a rapid development in more than twenty years. Therefore, it is necessary to strengthen the insurance investment system as well as the regulatory environment. IIIs investment in China's largest real estate company, Vanke Group, drew the attention and debate of insurance institutional investors throughout China. Holdings in insurance funds were traditionally deemed as "barbaric funds," and were not desired by corporations. The communities' dreadful imprint on IIIs has yet to be erased. Therefore, this article uses China as an example to investigate the impact of IIIs' stock ownership on firm value. This can provide proposals for resolving difficulties and issues, and serve as a reference for other countries around the world, particularly emerging economies with insufficient insurance systems.

***Reviewer’s Comment (Major Comment 2-(3)):***

*（3）Why is it important to analyse the impact of institutional insurance investors on value selection and creation and no other types of investors? What differentiates them from other investors with respect to these issues?*

**Authors’ Response (Major Comment 2-(3)):**

Thanks for the comment. Because the original presentation of the peculiarities of insurance institutional investors is incomplete, readers may wonder why the article favours insurance institutional investors above other institutional investors. In the first paragraph of the introduction, we describe the differences between insurance institutions and other institutional investors in light of the reviewers' questions. The main reasons for focusing on insurance institutional investors rather than others are that insurance assets make up a large percentage of total financial assets; insurance funds have their own unique properties; and insurance institutional investors' holding shares in leading Chinese real estate companies has caused a lot of controversy. The following new content has been added to the first paragraph of the introductory section (in blue):

Insurance institutions maintain a position that cannot be overlooked as key institutional investors in capital markets across the globe. According to the data of China insurance Yearbook，the insurance industry accounted for 17.8% of the global financial industry's total assets in the fourth quarter of 2019. US insurance institutions had $6.5 trillion in financial assets and held more than 25% of US corporate bonds in 2017 [28]. Insurance funds, unlike other institutional investors, are mostly debt funds fueled by policy obligations, rather than self-owned investment funds [45]. Thus, the size, horizon, duration, and risk of insurance funds are determined by the characteristics of the funds held, which must be matched with the insurance holders' liabilities [6]. Their impact on investee companies is likely to differ from that of other institutional investors due to these unique characteristics. The controversy over IIIs controlling the Chinese real estate company, Vanke Group over the nearly 3-year long period from 2015 to 2017, led to insurance funds being dubbed "barbaric funds" in China, and sparked interest and discussion about IIIs. This negative impression regarding IIIs have not yet been overcome. Regrettably, most extant academic circles conduct research on institutional investors as a whole, with few focusing on the specific impact of IIIs' shareholding on company value.

***Reviewer’s Comment (Major Comment 2-(4)):***

*（4）Are there any particularities that make their study interesting in terms of value selection and creation? As the introduction should be reviewed to strengthen motivation, similar changes should be considered in the conclusion to provide a more accurate picture of how your study makes a value-added contribution to the literature and how your study offers some practical implications.*

**Authors’ Response (Major Comment 2-(4)):**

Thanks for the comment. In response to the reviewer's suggestion, the revised manuscript has added research motivation and contribution to the literature in the introduction, and also included deeper thinking in the conclusion. The following is the modified part of the value-added contribution (first paragraph on p. 7):

The following aspects highlight the contributions of this paper: This paper undertakes a rigorous and in-depth analysis of the impact of IIIs' shareholding on company value and rectifies the absence of research on IIIs to some extent. Insurance companies were used as an example by Ge and Weisbach [28] to ascertain whether the financial health of institutional investors has an impact on their investment portfolios. However, despite insurers being major and influential investors, there is little research on the impact of institutional investors on firm value. This study investigates the value selection and creation functions of institutional investors' holdings on enterprises from both a theoretical and empirical perspectives, and thereby expands previous research on institutional investor heterogeneity.

Second, this paper examines the motivation and ability of insurance companies to create value from both positive and negative motivations, and the characteristics of insurance funds, insurance businesses, new accounting standards, and the impact of various stages of insurance company development on value creation. It examines numerous considerations and choices of insurance fund shareholding on a theoretical level and demonstrates the need for incorporating institutional heterogeneity in more in-depth research of institutional investors’ impact on company value. Theoretical analysis gives a reference point for businesses to assess III motivation.

Third, the value creation role is distinguished from an investment horizon in this study, and it thereby provides an empirical basis for regulatory authorities to encourage long-term holdings by insurance firms on a practical level. The "Vanke-Baoneng Battle" in the Chinese market from 2015 to 2017, which entailed the Baoneng Group's acquisition of a controlling interest in Vanke, a leading real estate company, has contributed to the perception of insurance funds as "barbaric funds." This article disproves the notion that insurance capital shares have a value-creating purpose and provides factual support for Chinese regulators' long-term investment strategy for insurance businesses. The empirical investigations serve as a foundation and point of reference for the creation of regulations. The focus of this article on the impact of Chinese insurance institutions' shareholdings on firm value can be useful as a benchmark for other nations, particularly emerging economies.

***Reviewer’s Comment (Major Comment 3-(1)(2)(3)):***

*3. Literature review, Theoretical Analysis and Hypothesis*

*This section is to some extent confusing. I see a lack of common thread, as each subsection does not link with the rest, and the hypotheses are very poorly justified. I suggest the following structure:*

*（1）A first subsection dedicated to insurance institutional investors, their conceptual isation, their main and most important differences with respect to other investors, their singular impact on company value selection and creation, etc. In my opinion, the inclusion of this subsection would be extremely important, as insurance institutional investors, as a concept, is not properly defined throughout the paper.*

*（2）A second subsection which reviews prior literature directly related to the topic, and explains how such studies give rise to the present work.*

*（3）A third subsection focusing on the effect of insurance institutional investors on company value selection and creation. This section should be devoted to providing more convincing and substantial arguments to justify how insurance institutional investors can affect company value selection and creation.*

**Authors’ Response (Major Comment 3-(1)(2)(3)):**

Thank you for the valuable comments. We agree wholeheartedly with the remarks on the paper's literature overview and theoretical analysis parts, as well as the specific revision suggestions. Following your suggestions, we discovered that the initial literature review and theoretical analysis had some flaws, such as a lack of correlation between paragraphs and a lack of rigor in the hypotheses argumentation process. The revised version integrates the literature review and theoretical analysis and deletes the material that is poorly connected to this paper and easily confused, after thorough reading and sorting, and combining the comments of the two reviewers. Furthermore, the most relevant literature is retained, and the latest relevant literature after 2019 is supplemented.

The revised content focuses on "Theoretical Analysis and Hypothesis" in the second part of the paper, and the specific structure has been adjusted.

The first section defines insurance institutional investors, explains features of insurance funds, then discusses the value selection function of insurance institutional investors and integrates the literature to show the differences between insurance institutions and other types of institutional investors. It examines the value selection function of insurance institutions at both the academic and practical levels, and proposes the first hypothesis of this paper.

The second part discusses the value creation function of fast-in and fast-out insurance institutional investors. This part begins with a literature review, summarizes the existing literature on institutional investors, and then examines whether there are any motivations or barriers to value creation for short-term equity-holding insurance institutional investors based on insurance institution characteristics. The second hypothesis of this paper is then proposed.

The third part discusses how long-term insurance institutional investors create value. This part begins with a literature review and then delves into the positive motivation and negative willingness of long-term insurance institutional investors to create value, reflecting the characteristics of insurance institutional investors more completely. Finally, competing hypotheses containing positive and negative views are proposed.

Each section of the revised literature review and theoretical analysis is structured as suggested by the reviewers. The structure is more defined, and the logical connections are more apparent. Please see the second half of the whole text, "Theoretical Analysis and Hypothesis," for the specific adjustments (marked in blue).

***Reviewer’s Comment (Major Comment 3-(4)):***

*（4）Finally, the research goals should be placed in the introduction section rather than in the Theoretical Analysis and Hypothesis section. As mentioned, this part should focus on providing stronger theoretical arguments to support the hypotheses under study.*

**Authors’ Response (Major Comment 3-(4)):**

We greatly appreciate for the suggestion. We should not set the research goal on "theoretical analysis and hypothesis," as the reviewer suggested. As a result, changes were made to the updated introduction and theoretical derivation. The introduction states the research questions and aims, and the "Theoretical Analysis and Hypothesis" section meticulously sources relevant literature for logical deduction and analysis. See the first two parts of the revised manuscript for more information.

***Reviewer’s Comment (Major Comment 4):***

4. Methodology

I encourage the author(s) to explain better why they have chosen the regression techniques used and not others. For example, why not SEM?

**Authors’ Response (Major Comment 4):**

Thanks for the comment. We fully agree with the reviewer's suggestion that the issue of model selection should be appropriately addressed. We've added an explanation of the model selection problem to Part V, "Model Regression Analysis and Counterfactual Testing," subsection (2), as requested. After processing, the data structure in this paper is a balanced panel data structure, and the Simultaneous Equation Model (SEM) is not used because the endogeneity of the model in this paper is primarily between the explained variables and the explanatory variables.That means there may be a reverse causal relationship between the shareholding ratio of insurance institutional investors and company value, but there is no reciprocal influence between explanatory variables in principle. Therefore, the instrumental variable approach and the lagged explanatory variable method can be used to solve this paper to a great extent. The endogeneity problem does not need the creation of system-wide simultaneous equations. The simultaneous equation model is not chosen in this paper, according to Aggarwal et al. (2011), Massa et al. (2013), and other literatures.

In the empirical regression, this paper selects the fixed effect model of panel data for regression and uses the instrumental variable method. Both LIML and 2SLS are used in the instrumental variable regression. The revised manuscript further explains why the GMM model was not chosen. The following are the precise modifications (first paragraph on p. 31):

The data generated in this paper is strongly balanced panel data (firm-year panel). We adopt a fixed effect model for model regression following Aggarwal et al. [1] and Massa et al. [48]. This is for a number of reasons: First, from an economic standpoint, omitted variables, such as corporate culture, executive characteristics, and so on, are often difficult to quantify, and "heterogeneous" differences in companies, which are difficult to observe and measure, frequently lead to serious endogenous problems. The fixed-effect model can lessen the endogenous problem produced by the bias interference of omitted variables as the differential process in the estimated approach of the fixed-effect model can eliminate "time-invariant" firm heterogeneity to some extent. Second, this paper used statistical analysis such as the F test and the Hausman test from a statistical standpoint. The results reveal that at a 1% significance level, the fixed effect model is the most appropriate. The author may provide F test data upon request, and the values of Hausman test statistics can be found in Table 4. The Two-Stage Least Squares (2SLS) estimation results are used in this paper for the long-term holding type and the fast-in-fast-out type; simultaneously, the Limited Information Maximum Likelihood estimation (LIML) method, which is insensitive to weak instrumental variables, is used to obtain the model parameters. The reasonableness of the instrumental variable selection is indirectly validated if the difference between the two estimations is minimal. Thirdly, we diagnose the model residuals and draw a scatter plot of residuals and model fitting values at the same time, demonstrating that the model has no heteroscedasticity problem. Similarly, since the Generalized Method of Moments (GMM) regression method is suitable for the case where the model has heteroscedasticity, the GMM estimation method is not used in this paper.

***Reviewer’s Comment (Major Comment 5):***

5. Empirical results

The authors should make a greater effort to find previous papers that confirms their results.

**Authors’ Response (Major Comment 5):**

Thanks for the comment. We carefully examined the descriptions of the empirical results in various references after being reminded by the reviewer. Following previous literature, we have improved the explanation of this paper's empirical results, as mentioned in the revised manuscript's "Descriptive Statistics" and "Model Regression Analysis and Counterfactual Testing" sections (marked in blue).

***Reviewer’s Comment (Major Comment 6):***

6. Conclusions

In this section, I really missed a deep reflection on the part of the author(s). As researchers, our work should reflect our ability to think and not just describe results. This paper reveals interesting results, and the importance of these results is not adequately reflected in this section. It would be interesting, for example, to relate the obtained results more closely to previous literature and to point out more explicitly the contributions of the study in comparison to the previous literature. In addition, it is very important to elaborate further on the contributions and practical implications, as well as to indicate some limitations and future avenues of research may help to guide future researchers in this field and improve the quality of the manuscript. For example, this section could also be strengthened by further detailing the practical implications of the study, e.g., for Chinese companies.

**Authors’ Response (Major Comment 6):**

Thanks for the comment. We've made a number of changes and enhancements based on your feedback. We examine the paper's findings in greater depth and clarify the paper's incremental contribution to the current research. We highlight the article's contributions and practical ramifications, as well as provide more specific and useful recommendations for regulators and stakeholder companies. We indicate the study's limitations, flaws, and future research directions.

***Reviewer’s Comment (minor issue 1):***

Other minor issues

There are no references to PLOS ONE. Thus, I suggest authors to include some article from PLOS ONE to underline the importance of the topic for the journal.

**Authors’ Response (Minor issue 1):**

Thank you so much for your suggestions. We have supplemented the literature in PLOS ONE relevant to the study in the bibliography in response to the reviewer's suggestion. For details, see added references [34] and [38] in the references section.

# Responses to Reviewer2:

Our sincere gratitude to the reviewer for the constructive comments on our manuscript. Your constructive comments on the paper were extremely valuable in improving the quality of the paper. We have carefully revised the manuscript and adopted all of your suggestions. In what follows, your comments are shown in italics and followed by our point-by-point responses. All the page numbers refer to those in the revision. Changes made according to the Major Comments in the revised manuscript are highlighted in blue color of the revised paper for ease of your reference. We have made numerous minor changes throughout the paper, according to your special comments. We believe that you will find this report and our revised manuscript have satisfactorily addressed the issues you have raised and that the revised manuscript meets the high standards of PLOS One. We thank you in advance for your valuable time in reviewing the manuscript.

**Main Comments**

*The whole paper needs a English grammar review.*

***Reviewer’s Comment (Major Comment 1-(1)):***

*1. INTRODUCTION*

*I miss in this section the following aspects: gap in the literature that your paper aims to fill in, your contribution and findings. In this way, I think it is necessary to distinguish between this topic worldwide and specifically in China.*

*I suggest restructuring Sections “Introduction”, “Literature Review” and “Theoretical Analysis and Hypothesis”.*

**Authors’ Response (Major Comment 1-(1)):**

We appreciate for the suggestion. The "Introduction," "Literature Review," and "Theoretical Analysis and Hypothesis" have all been reorganized. The present introduction is structured as follows, with clear writing for each component suggested by reviewers, and the new introduction is shown in the blue in the revised manuscript.

The first paragraph discusses the significance and peculiarity of insurance funds, as well as the issues that arise from their holdings in practise. This is our motivation for writing this article.

The second paragraph summarizes our knowledge of the research issue. It is noted that there are very few research on the heterogeneity of institutional investors in the available literature, with a clear lack of attention paid to insurance institutional investors.

The third paragraph lists the research gaps that our study addresses.

The fourth paragraph contains research objectives as well as a brief summary of how we plan to attain them. There's also a reason why we chose to conduct research on China's capital market or insurance institutional investors.

The fifth paragraph introduces the article's research findings and contributions to the literature.

We completely agree with the reviewer's suggestions for restructuring the "Introduction," "Literature Review," and "Theoretical Analysis and Hypothesis" sections. Following the reviewer's suggestion, we discovered that the existing literature review and theoretical analysis have several flaws, such as a lack of correlation between paragraphs and a lack of rigour in the hypotheses argumentation process. The revised version integrates the literature review and theoretical analysis, and deletes the material that is poorly connected to this paper and easily confused, after thorough reading and sorting, and combining the comments of the two reviewers. Furthermore, the most relevant literature is retained, and the latest relevant literature after 2019 is supplemented. The structure of each part is clearer and the logical relationship is closer after the adjustment. Please see the second half of the full text, "Theoretical Analysis and Assumptions," for further information on the revision.

***Reviewer’s Comment (Major Comment 1-(2)) :***

*From lines 50 to 54 I think you need to add citations to support those statements.*

**Authors’ Response (Major Comment 1-(2)) :**

Thank you for suggesting that the content stated in lines 50 to 54 of the original manuscript, i.e. “On the other hand, the insurance fund has its unique properties in terms of capital source, risk tolerance, investment philosophy, etc. Its impact on the company value may be different from the institutional investors, which makes the research on the influence of insurance institutional investors’ holding on the company value more significant in value”, be cited. We've included literature and associated news reports to the amended introduction, based on the reviewer's recommendation, to better support *those statements*. The characteristics of the insurance funds are described in the first paragraph of the revised introduction (marked in blue).

***Reviewer’s Comment (Major Comment 1-(3)) :***

*The justification of your paper, lines 60-61 is quite weak.*

**Authors’ Response (Major Comment 1-(3)):**

Thank you to the reviewer for pointing out that the introduction is weak in argumentation. It is indeed incorrect for the original text to make the claim that "the impact of institutional investors on companies has become an urgent problem in academic research and practice" without a solid foundation for arguments. We restructured the material of the introduction and rewrote it to clarify the paper's research background, the significance of the research, the research objectives, the research viewpoints, and the research contributions. The structure of the existing introductory section is described in **Authors’ Response (Major Comment 1-(1))**.

***Reviewer’s Comment (Major Comment 2-(1)):***

*2. LITERATURE REVIEW / THEORETICAL ANALYSIS / HYPOTHESES*

*As stated before, I believe you should restructure this section, linking the review of the literature to the hypothesis. Indeed, the hypotheses should be right after the literature review of each of them, as usual. There are a lot of information, but it is disconnected and its reading is quite messy, and sometimes, apparently, contradictory. You show the arguments in the literature in favour and against the impact of insurance institutional investors on companies’ return. However, you do not take a position in your paper. Moreover, your review looks a kind of disconnected from the hypotheses. That is why I suggest restructuring the three first sections.*

**Authors’ Response (Major Comment 2-(1)):**

Thank you for the valuable comments. We agree wholeheartedly with the remarks on the paper's literature overview and theoretical analysis parts, as well as the specific revision suggestions. Following your suggestions, we discovered that the initial literature review and theoretical analysis had some flaws, such as a lack of correlation between paragraphs and a lack of rigor in the hypotheses argumentation process. The revised version integrates the literature review and theoretical analysis and deletes the material that is poorly connected to this paper and easily confused, after thorough reading and sorting, and combining the comments of the two reviewers. Furthermore, the most relevant literature is retained, and the latest relevant literature after 2019 is supplemented.

In the second half of the paper, the revised content concentrates on "Theoretical Analysis and Hypothesis." The hypothesis and the literature review are inextricably linked. Each time a point of view is presented, a hypothesis is proposed to help clarify the logic and hierarchy. For the structure of the second part, "Theoretical Analysis and Hypothesis," we made the following changes and rearrangement:

The first section defines insurance institutional investors, explains features of insurance funds, then discusses the value selection function of insurance institutional investors and integrates the literature to show the differences between insurance institutions and other types of institutional investors. It examines the value selection function of insurance institutions at both the academic and practical levels, and proposes the first hypothesis of this paper.

The second part discusses the value creation function of fast-in and fast-out insurance institutional investors. This part begins with a literature review, summarizes the existing literature on institutional investors, and then examines whether there are any motivations or barriers to value creation for short-term equity-holding insurance institutional investors based on insurance institution characteristics. The second hypothesis of this paper is then proposed.

The third part discusses how long-term insurance institutional investors create value. This part begins with a literature review and then delves into the positive motivation and negative willingness of long-term insurance institutional investors to create value, reflecting the characteristics of insurance institutional investors more completely. Finally, competing hypotheses containing positive and negative views are proposed.

Each section of the revised literature review and theoretical analysis is structured as suggested by the reviewers. The structure is more defined, and the logical connections are more apparent. Please see the second half of the whole text, "Theoretical Analysis and Hypothesis," for the specific adjustments (marked in blue).

The revised literature review and theoretical analysis have a more logical relationship and a clearer framework. Please see the second part of the revised manuscript "Theoretical Analysis and Hypothesis (marked in blue) for more details.

***Reviewer’s Comment (Major Comment 2-(2)) :***

*In line 73 you mention “information disclosure”, however I cannot see the link of this concept with your paper.*

**Authors’ Response (Major Comment 2-(2)) :**

Thanks for your detailed and specific comments. In line 73 "Most scholars studied that there would be a limitation if the overall institutional investors could improve the quality of corporate information disclosure (Jensen and Mecling, 1979; Shleifer and Vishny, 1986)", we cited two papers, which are Jensen and Mecling (1979) and Shleifer and Vishny (1986), respectively.

We carefully read two literatures again. In the relevant remarks of institutional investors, Jensen and Mecling (1979) believed that that groups such as institutional investors and securities analysts have advantages in corporate supervision activities, and their degree of specialization is higher. However, the "improve the quality of corporate information disclosure" stated in this paper is not directly reflected in the literature, so we have removed the reference to Jensen and Mecling (1979) following revision. Another paper, Shleifer and Vishny (1986), discovered that companies with a higher percentage of shareholders overseeing them perform better. In the revised manuscript, we continue to mention Shleifer and Vishny (1986).

Please refer to the relevant content in the introduction part of the revised manuscript, as the literature review in the section on institutional investors and corporate value has been moved to the introductory part (last paragraph on p. 3 marked in blue):

The available literature on institutional investors' role can be divided into three groups. Institutional investors, according to one viewpoint, will actively participate in corporate governance, and thereby effectively boosting company performance and value [46, 49, 58, 62]. According to this view, institutional investors can effectively supervise the company's investment operations [59], improve the quality of earnings [15, 16, 50, 67], or cut agency costs by overseeing the behavior of major shareholders and executives of publicly traded companies [42, 47, 53, 58], which improve company performance and increase corporate value, as they have the advantages of abundant capital, high shareholding ratio, abundant information channels, and strong investment and research teams [74].

***Reviewer’s Comment (Major Comment 2-(3)) :***

*In line 131 is the first time you use the acronym CSR, and it is necessary to explain what these words stand for.*

**Authors’ Response (Major Comment 2-(3)) :**

Thanks for the comment. We have carefully checked all the abbreviations and their corresponding explanation.

***Reviewer’s Comment (Major Comment 2-(4)) :***

*Citation in line 143 is wrong.*

**Authors’ Response (Major Comment 2-(4)) :**

Thanks for the correction regarding Line 143. We have carefully checked all the citation.

***Reviewer’s Comment (Major Comment 2-(5)) :***

*In lines 193-194 you state that you contribute to the literature analysing positive and negative motives, but I miss them in your paper.*

**Authors’ Response (Major Comment 2-(5)) :**

Thank you for the comments. We also agree that the original manuscript does not make the line between "positive and negative motivation" clear enough. The revised manuscript clearly analyses the impact of long-term shareholding insurance institutional investors on company value from the two perspectives of "positive" and "negative" motivations; for more information, see the third subsection, i.e. "The Value-Creating Function of Long-term Equity-Holding Insurance Institutional Investors" of the second part, i.e. "Theoretical Analysis and Hypotheses," of the manuscript. The following is an excerpt (marked in blue) of the content:

The impact of insurance institutions' long-term shareholding on company value is potentially two-sided. Insurance companies have a vested interest in creating value. First, the long-term holding demand of insurance companies and the poor risk tolerance of debt management push them to participate in corporate governance, supervise executives, reduce business agency expenses, and promote corporate value improvement to reap the long-term benefits. Liu et al. [45] evaluated the shareholding characteristics and preferences of insurance organizations and observed that IIIs have a long-term value investing philosophy, which is beneficial to a stable capital market. Second, the healthy development of insurance companies requires support from non-insurance businesses both upstream and downstream. Banks play a key role in the marketing of insurance goods as a part-time insurance agency channel. Insurance companies listing banks will have an impact on bank decision-making, allowing insurance businesses to benefit from synergies. The pharmaceutical and biological industries are inextricably linked to the cost and quality of medical insurance and critical illness insurance provided by insurance companies. The real estate sector is intertwined with the insurance industry's pension community, as well as pension and long-term care insurance. Furthermore, insurance firms are expected to offer not only current and future pensions, but also limited pension services that can be purchased by pensioners.

The auto sector is inextricably linked to insurance firms' auto insurance businesses. Insurance companies are expanding their industrial chain in sectors such as pre-sale, after-sale maintenance, daily maintenance, roadside assistance, and vehicle finance. Insurance companies are willing to offer more diverse products and services to their customers, which can increase customer contribution value; insurance companies make full use of the synergies among their various businesses in terms of brands, channels, funds, and so on, which can not only expand the customer base but also increase customer stickiness through cross-selling. To summarize, insurance companies are the final payers in a variety of industries, with a large client base and on-the-ground teams. They have inherent advantages in terms of location as well as significant economic benefits in terms of increasing business boundaries along the industry chain. As a result, we believe that insurance businesses have the desire to create value to benefit from strategic synergy. Third, the new accounting standard IFRS9（International Financial Reporting Standards）, has been fully implemented from 2021, and insurance companies are eager to boost long-term equity investment to reduce profit volatility. Equity assets must be valued at fair value under the new standard, and equity changes will be directly reflected in the income statement, and thus have a rather considerable influence on net profit. According to IFRS9, investments holding more than 5% of a company and a director seat can be included in the long-term equity investment account, which is accounted for using the equity method, so that investment performance is not affected by stock price fluctuations and is more stable. This has an impact on the evolution of insurance fund investment behavior toward long-term value generation to some extent.

Long-term IIIs, on the other hand, have negative incentives to build value, as creating value for companies necessitates an investment of time and money. Since insurance funds are commercial capital, they have no obligation to produce value for other businesses. The benefits of value creation does not always outweigh the expenses. Second, insurance companies are "stress-sensitive" institutional investors [12], as insurance companies and investee companies have commercial links [21], such as offering insurance services [75]. They are concerned that their own oversight and involvement in the enterprise's management may result in the loss of existing or potential commercial contracts and impact their own income. Therefore, the cost of performing its supervisory function is higher for insurance companies than for other pressure-resistant institutional investors (funds, social security funds, QFII, enterprise annuities), and it lacks the motivation to actively participate in corporate governance [43], making it difficult to contribute to the creation of value for businesses. Third, the insurance industry's development and asset management are still in their infancy; there are variances in the development of insurance institutions, and not every insurance business is at the stage of expanding the insurance industry chain. Short-sighted behavior and the objective development stage are highly likely to result in no value generation function.

Based on the above analysis, this paper proposes a third hypothesis:

Research hypothesis H3a: Investors in long-term holding type insurance institutions have the function of value creation, which means that increases in their shareholding have a positive impact on the company's value changes.

Research hypothesis H3b: Long-term holding type insurance institutional investors do not have the function of value creation, i.e., shareholding changes of long-term holding type insurance institutional investors have no substantial association with changes in the company's value.

***Reviewer’s Comment (Major Comment 2-(6)) :***

*In lines 216-217 you need to use citations to support such a strong assumption.*

**Authors’ Response (Major Comment 2-(6)) :**

Thanks for the suggestion with respect to Line 216-217: “Whether short-term or long-term, insurance companies want to invest in companies with rising value, not the other way around. There is no doubt about this will. As for this point, we will not carry out the theoretical analysis.” We completely agree with the reviewer that the value selection function lacks sufficient theoretical justification. In the second part of the paper, we incorporated a rather full theoretical analytical procedure to the value selection function of insurance institutional investors. Please see the first subsection "The Value Selection Function of III" in the "Theoretical Analysis and Hypotheses" section of the revised manuscript for further information. The following is the amended content:

Theoretical Analysis and Hypotheses

The Value Selection Function of III

Insurance firms and insurance asset management businesses that invest in stocks are referred to as IIIs. The primary source of funds for IIIs is debt funds derived from policy liabilities. When holding shares, IIIs must assess if investment returns can sustain the operation of insurance businesses [28]. That is, the return on investment should be in line with the needs for profitability, liquidity, and safety. Consequently, whether it is short-term stock holding for profit or long-term stock holding to meet liabilities, insurance institutions must choose firms with investment value. Many studies have revealed that institutional investors, including IIIs, have the ability and inclination to choose value. Studies such as Aggarwal et al. [1], Li and Li [41], Mu and Zhang [52], Shi and Li [57], and Wu and Jiang [,71] have demonstrated that ratio of institutional investors' shareholding, and indicators like firm performance and market value have a substantial positive link. According to Wang and Ge [69], the shareholding ratio of life insurance businesses is substantially connected with the operating success of publicly traded companies. Wen and Feng [70] pointed out that securities’ investment funds are under pressure to frequently publish investment results while discussing the relationship between heterogeneous institutions and independent innovation. However, as insurance funds are exempt from disclosing their investment portfolios on a regular basis and from participating in general fund rankings, they are incentivized to seek out more creative businesses to invest in. The ability of insurance institutions to select value is very clearly demonstrated in practice. The real estate industry, which was traditionally seen as a safe investment by insurance capital in China, was quickly abandoned by the end of 2021 as the real estate environment deteriorated and affected revenue. IIIs considered real estate businesses as "sweet pastry" in the past because of their high return on equity (ROE) and low volatility, and features such as value and growth. The insurance industry also intersects with the real estate market in terms of pension real estate. However, IIIs withdrew their money rapidly when the period of huge payouts in the real estate business ended. Therefore, choosing organizations that are worthy investments is an unavoidable requirement for insurance companies. In addition, insurance funds in China's capital market are huge in scale, have a lot of information channels, and have good screening skills [74]. This aids in value selection. Insurance institutions' shareholding scale continues to rise in the Chinese market, with the percentage of investments in the A-share circulating market rose from 3.6% in 2016 to 5.45 percent in 2018. The amount of money that insurance funds invested in stocks and securities accounted for 12.39 percent of the total at the end of the first quarter of 2019. This growth has been fueled in part by the China Banking and Insurance Regulatory Commission's suggestion to insurance funds to grow their equity holdings in publicly traded companies. Thus, insurance institutions have a great screening power to select desirable enterprises for investment based on government assistance and their own strength.

Based on the above analysis, this paper proposes the following first hypothesis:

Research hypothesis H1: IIIs with long-term or short-term shareholding have the ability to choose value, that is, the shareholding ratio of IIIs has a significant positive impact on company value.

***Reviewer’s Comment (Major Comment 2-(7)) :***

*Lines 222-223 and 262 seem to be contradictory, as line 254 and 257-258.*

**Authors’ Response (Major Comment 2-(7)) :**

Thanks for the comment. Lines 222-223 and 262 analyze insurance institutions' incentive to actively create value after holding shares, while lines 254 and 257-258 analyze their propensity to passively create value after holding shares.

The duality of positive and negative motivation in the original version was not clear enough. In the revised version, we explicitly present an analysis of both motivations in the second contribution of the Introduction section, i.e., “Second, this paper examines the motivation and ability of insurance companies to create value from both positive and negative motivations, and the characteristics of insurance funds, insurance businesses, new accounting standards, and the impact of various stages of insurance company development on value creation. It examines numerous considerations and choices of insurance fund shareholding on a theoretical level, and demonstrates the need for incorporating institutional heterogeneity in more in-depth research of institutional investors’ impact on company value. Theoretical analysis gives a reference point for businesses to assess III motivation.”

In addition, in the revised version, in the section " The Value-Creating Function of Long-term Equity-Holding IIIs", we specifically analyze insurance institutions' desire to actively create value after holding shares in listed companies and the reasons for negative participation in value-creating activities in the view of the characteristics of insurance funds. Thus, alternative hypotheses have been proposed. Please see the third subsection, i.e. "The Value-Creating Function of Long-term Equity-Holding IIIs", of "Theoretical Analysis and Assumptions" for more information (marked in blue). For an excerpt, please see Authors' Response (Major Comment 2-(5)).

***Reviewer’s Comment (Major Comment 2-(8)) :***

*In line 276, in your H1, suddenly, you use the term FIFO. You need to explain this concept and how it is connected to the literature and the aim of you paper before stating the hypothesis.*

**Authors’ Response (Major Comment 2-(8)) :**

Thanks for the comment. To make the presentation more precise, we carefully changed the abbreviation after clarifying the meaning of FIFO in the main text. At the same time, we updated all the same unspecified meanings of abbreviations in the process of revising the manuscript, based on the reviewer's suggestions.

The following is an excerpt of the relevant content: (the explanation part is marked in blue on p. 10):

The Value Creation Function of FIFO IIIs

The question of whether IIIs have a value creation function needs to be investigated further, and any discussion of this topic must take into account the holding term of IIIs. Institutional investors may have various long- and short-term goals, concerns, and effects on firm value. In this article, institutional investors who hold short-term equities are referred to as FIFO institutional investors, which signifies that these funds withdraw their money from stocks after a short period of time. Many studies suggest that institutional investors with short-term stock holdings are less motivated to conduct corporate monitoring tasks, and that they anticipate corporate agents to prioritize present success over long-term value, resulting in them being value selectors rather than value producers [1, 12]. Thus, for example, management may cut R&D expenditures in a firm with a high proportion of short-term institutional investors' holdings to avoid a decrease in the stock price [9]. Therefore, FIFO investors take on the role of short-term speculators, and often take the negative option of "voting with their feet" if they are displeased with the company's performance [54].

***Reviewer’s Comment (Major Comment 3):***

*MODEL DESIGN AND VARIABLES*

*I strongly encourage you to re-write “Main Model Design” in a clearer way. It is difficult to understand your models.*

**Authors’ Response ：**

We have made changes in response to your suggestions. The following are the major changes:

(1) The rationale for employing the lag period in the model is explained.

(2) The details not explained in the original version have been supplemented and improved.

(3) Each of the reviewer's questions was amended and improved one by one.

***Reviewer’s Comment(Major Comment 3-(1)):***

*In line 294, what is “issue t“? It means time, but you use different words.*

**Authors’ Response (Major Comment 3-(1)):**

Thanks for your correction. In Line 294 of the original manuscript, we wrote “$\mathrm{ComValue}_{\mathrm{jit}}$ represents the value of listed company i in period t; $\mathrm{Shareh}_{\mathrm{jit}}$ is the shareholding ratio of an insurance company to listed company i in period t”. In the revised version, all variables that were not properly stated have had their meanings added. We added an additional description in "Model Design and Variables" for the description of t representing time, and utilised quarter t (t=1,2,...25) to replace period t in the original manuscript. The following is an excerpt from the text (the description about t is in red, and the corrected text is in blue):

Given that IIIs' decision to buy stocks in period t is based on the company's value performance in period t and before, and that IIIs' ability to choose value is reflected in the company's performance after period t (where t represents time; since the data used in this article spans quarters, t represents a quarter, where t=1, 2, 3.... 25)…

***Reviewer’s Comment(Major Comment 3-(2)):***

*In line 296, “control variables” instead of “control variable”.*

**Authors’ Response (Major Comment 3-(2)):**

Thanks for your correction. This mistake has been fixed across the manuscript.

***Reviewer’s Comment(Major Comment 3-(3)):***

*Why are you using “L”, “L2” and “D”? Explain the economic reasoning and connect it to the hypotheses.*

**Authors’ Response (Major Comment 3-(3)):**

Thanks for your suggestion. First, we defined the meanings of L. and D. in the revised "Main Research Model Design" section; second, we included why we need to utilise lag and difference variables in this section, and the introduction of each model is combined with the relevant assumptions. The following is an excerpt from the amended text:

Main Research Model Design

Given that IIIs' decision to buy stocks in period t is based on the company's value performance in period t and before, and that IIIs' ability to choose value is reflected in the company's performance after period t (where t represents time; since the data used in this article spans quarters, t represents a quarter, where, t=1, 2, 3.... 25). Furthermore, there may be a strong relationship between insurance institutions' shareholding ratio and company value: insurance institutions' participation in corporate governance may promote better value performance of the company; and companies with high value are more likely to be favored by insurance institutions, who then take stakes in the company. This suggests that, to some extent, the shareholding ratio of insurance institutions and firm valuation are causally related, which could cause endogeneity issues in the model. Based on the two reasons above, a more reasonable model to evaluate the value selection function of insurance institutions is to utilize the t-period III's shareholding to regress the company value in t+1 period.

Based on the foregoing assumptions, we use the business value as the explained variable and the shareholding ratio of IIIs with a lag period as the explanatory variable to evaluate the first hypothesis of this study, namely, whether IIIs have the role of value selection. In this paper, the value selection model is created using the following linear regression model:

$$\mathrm{ComValue}_{\mathrm{jit}}=\beta_{j1}*{L.Shareh}_{\mathrm{jit}}+\beta_{j2}*\mathrm{Control}_{\mathrm{jit}}+\alpha_{\mathrm{ji}}+\vartheta_{\mathrm{jt}}+\varepsilon_{\mathrm{jit}} \left( A \right)$$

where $\mathrm{ComValue}_{\mathrm{jit}}$ is the value of listed company i in period t; $\mathrm{Shareh}_{\mathrm{jit}}$ is the shareholding ratio of listed company i in period t; $\mathrm{Control}_{\mathrm{jit}}$ is the control variable; $\alpha_{\mathrm{ji}}$ and$\vartheta_{\mathrm{jt}}$ are the sample companies’ individual and time effects, respectively; and$\varepsilon_{\mathrm{jit}}$ is random error. Furthermore, j =1, 2, with 1 denoting long-term stock holding IIIs, and 2 denoting FIFO IIIs. L. in model(A) represents the first-order lag operator, and the one-period lag is used because the proof of the value selection ability of IIIs requires a certain period of time to test, which has a lag; however, the endogeneity problem can be solved to a certain extent. The value selection model (A) is primarily utilized to see if the IIIs presented in Hypothesis 1 have a value selection function.

We use the change in company value (${D.ComValue}_{\mathrm{jit}}$) as the explanatory variable and the stock holdings of IIIs as the explained variable to test the second and third hypotheses of this paper, namely, whether IIIs have a value-creating function. The change in the shareholding ratio of insurance institutions (${D.Shareh}_{\mathrm{jit}}$) is employed as an explanatory variable in the value creation model that follows (B):

$${D.ComValue}_{\mathrm{jit}}=\gamma_{j1}*{D.Shareh}_{\mathrm{jit}}+\gamma_{j2}*\mathrm{Control}_{\mathrm{jit}}+\alpha_{\mathrm{ji}}+\vartheta_{\mathrm{jt}}+\varepsilon_{\mathrm{jit}} \left( B \right)$$

What needs to be clarified in model (B) is the meaning of D., which stands for the first-order difference operator, especially:

$${D.ComValue}_{\mathrm{jit}}=\mathrm{ComValue}_{\mathrm{jit}}-\mathrm{ComValue}_{jit-1}$$

$${D.Shareh}_{\mathrm{jit}}=\mathrm{Shareh}_{\mathrm{jit}}-\mathrm{Shareh}_{jit-1}$$

Model (B) is utilised as a model for evaluating value creation because even if Model (A) is constructed, it can only determine the value selection function of IIIs; however, determining whether IIIs have a value creation function is challenging. Model (B) must establish the explained variable as a difference operator to observe the improvement of the company's value to test if the III's shareholding may help enhance the company's worth. Changes in the shareholding ratio of insurance institutions are also used as explanatory variables in model (B), primarily for two reasons: Changes in insurance institutions' shareholding ratios can reflect their preferences at a given time - buying or selling stocks is an important way for insurance institutions to participate in corporate governance and influence the value of companies, referred to as "voting with hands" and "voting with feet".

This paper determines the indicators to measure the company's value after establishing the models (A) and (B). Most studies, like those by Cornett et al. [18], Ferreira and Matos [26] and Healy [32], employ Tobin's Q [33] value to evaluate firm value, but China's stock market's price discovery function is in a nascent stage, and there is a strong speculative atmosphere. It is difficult to determine the true worth of publicly traded companies based on Tobin’s Q [33]. Therefore, this article selects three measures to estimate the value of organisations after meticulous screening. Our first indicator, based on Chaganti and Damanpour [11] and Tang and Song [66], chooses earnings per share (EPS) as the key explanatory variable. Second, two alternative proxies, return on assets (ROA) [18, 36] and return on equity (ROE) [41, 63], are utilised as robustness test indicators. The parameters $\beta_{j1}$ and $\gamma_{j1}$ test the value selection and value creation functions of IIIs, respectively, where j as mentioned previously, takes 1 for long-term shareholding IIIs, and 2 for FIFO insurance institutional investment. The estimated parameters $\beta_{11}$ and $\beta_{21}$ are expected to be positive under assumption H1, and $\gamma_{21}$ is expected to be negative or insignificant under assumption H2, implying that FIFO IIIs do not have the function of value creation; the parameter $\gamma_{11}$ is expected to be positive under assumption H3a, but $\gamma_{11}$ is expected to be negative or insignificant in H3b.

***Reviewer’s Comment(Major Comment 3-(4)):***

*It is necessary to support with citations the different dependent variables shown in this section.*

**Authors’ Response (Major Comment 3-(4)):**

Thanks for your comment. This paper includes citations to the references of the model variables in both the revised model design section and the data section to guarantee academic rigour, as suggested by the reviewer. Below is a portion of the material that has been excerpted (marked in blue):

“This paper determines the indicators to measure the company's value after establishing the models (A) and (B). Most studies, like those by Cornett et al. [18], Ferreira and Matos [26] and Healy [32], employ Tobin's Q [33] value to evaluate firm value, but China's stock market's price discovery function is in a nascent stage, and there is a strong speculative atmosphere. It is difficult to determine the true worth of publicly traded companies based on Tobin’s Q [33]. Therefore, this article selects three measures to estimate the value of organisations after meticulous screening. Our first indicator, based on Chaganti and Damanpour [11] and Tang and Song [66], chooses earnings per share (EPS) as the key explanatory variable...”

“Descriptive Statistics

The explained variable is the firm value (expressed by EPS, ROA, and ROE), and according to Cornett et al. [18], the key explanatory variable is the shareholding ratio of IIIs. Table 1 shows the detailed definitions. The control variables in the model include: (1) Corporate governance structure (Conc), which is measured by the company's ownership concentration. According to previous research literature, firm value does not alter ownership structure, and hence there is no reverse causal relationship between ownership structure and company value [25, 31, 66, 76]. The mutual interference between the model residual and the ownership structure can be moderately reduced if the control variable includes the ownership structure index, which improves the effectiveness of the institutional investor ownership concentration (Insfir3, Insfir5) as an instrumental variable. (2) Company growth (Grow), following Aggarwal et al. [1], this work controls for the company's growth as it may have an impact on its value. (3) Financial leverage (AssLia). An asset-liability ratio is selected to measure the impact of financial leverage as financial leverage has an influence on firm value [23, 65]. (4) company size (Size). The impact of company size is factored into the model as a great number of studies have shown that company size has an impact on the company's value. (5) Institutional investor heterogeneity (NonIns). The characteristics of shareholders impact firm value, according to Huang et al. [33], Song et al. [60], and Tang and Li [65], thus, this article addresses the impact of non-IIIs. (6) The lag term (PreValue) of the company's value measurement variable is also measured as it may be more difficult to enhance the performance of listed firms with high value. Thus, the previous period's company value is introduced as a control variable to models (A) and (B) to mitigate the endogenous problem [66, 14]. Higher-order lag terms (L3.ComValue and L4.ComValue) are likewise considered as instrumental variables [3, 14].”

***Reviewer’s Comment(Major Comment 3-(5)):***

*Regarding the endogeneity problem, in my case, it is the first time that I meet models like yours to deal with this potential problem. I believe either you strongly support your models (from A to D) with the literature or use the well accepted (system)* ***GMM approach****, which uses most of all the available moments (lags) for the independent and control variables. In your case, you only use the second lag (L2). I believe that it could be useful to add in Table 2 and 3, variables used in your paper such as: InsFir3, InsFir5, InsDura.*

**Authors’ Response (Major Comment 3-(5)):**

Thanks for the comment. After reviewing this paper and the literature in depth, we discovered that the introduction of the model design process was unclear, resulting in a discrepancy between the endogeneity method's description and the real scenario. As a result, we added a second description of the endogeneity treatment approach and clarified why we didn't use the GMM method. To cope with the endogeneity problem, we primarily focus on two objectives in this paper. One is to lag the explanatory variables by one period. Refer to the literatures of Fields (1979), Cornett (2007) and Aggarwal et al. (2021); the second is to choose instrumental variables. The proportions of the invested company's top three and top five institutional investors are the instrumental variables (that is, the specific equity concentration, recorded as InsFir3 and InsFir5 respectively). The selection is primarily based on Demsetz's (1983) and other scholars' equity irrelevance hypothesis. This instrumental variable was also used by (Li Lei and Han Liyan, 2014). At the same time, this paper follows Arellano and Bond (1991) and Cheng et al. (2011) in using the higher-order lag term's explained variable (L3.ComValue and L4.ComValue) as an instrumental variable. Indeed, throughout the empirical process, we only used InsFir3, InsFir5, L3.ComValue, and L4.ComValue as instrumental variables). The following is the main revision regarding this comment (modified parts are marked in blue):

“**Main Research Model Design**

Given that IIIs' decision to buy stocks in period t is based on the company's value performance in period t and before, and that IIIs' ability to choose value is reflected in the company's performance after period t (where t represents time; since the data used in this article spans quarters, t represents a quarter, where, t=1, 2, 3.... 25). Furthermore, there may be a strong relationship between insurance institutions' shareholding ratio and company value: insurance institutions' participation in corporate governance may promote better value performance of the company; and companies with high value are more likely to be favored by insurance institutions, who then take stakes in the company. This suggests that, to some extent, the shareholding ratio of insurance institutions and firm valuation are causally related, which could cause endogeneity issues in the model. Based on the two reasons above, a more reasonable model to evaluate the value selection function of insurance institutions is to utilize the t-period III's shareholding to regress the company value in t+1 period.”

**“Selection of Instrumental variables**

...Therefore, alternative indicators for instrumental factors must be sought. This paper uses the institutional investor equity concentration of sample companies as a tool variable, that is, the proportion of the top three and top five institutional investors in the invested company (the specific equity concentration recorded as InsFir3 and InsFir5) as an instrumental variable, based on the relevant research results of Demsetz [19] and Demsetz and Lehn [20] in the field of corporate governance and referring to the idea of selecting instrumental variables by Hansen et al. [39]. Simultaneously, the higher-order lag term (L3.EPS and L4.EPS) of the insurance company's shareholding ratio is used as an instrumental variable to mitigate the problem of weak instrumental variables [3].”

“**Descriptive statistics**

(6) The lag term (PreValue) of the company's value measurement variable is also measured as it may be more difficult to enhance the performance of listed firms with high value. Thus, the previous period's company value is introduced as a control variable to models (A) and (B) to mitigate the endogenous problem [66, 14]. Higher-order lag terms (L3.ComValue and L4.ComValue) are likewise considered as instrumental variables [3, 14].”

This is a more extensive supplement and description of our endogeneity section, which is also supplemented in the model selection portion of this study. The data in this research has a fully balanced panel data structure. The fixed effect regression approach is used to analyse the data in this research. In the original manuscript, the reasons for using fixed effects are also described in length. The revised manuscript adds to the mentioned literature and explains why GMM estimate is not used. For further information, see "Model Regression Analysis":

“Model Regression Analysis

The data generated in this paper is strongly balanced panel data (firm-year panel). We adopt a fixed effect model for model regression following Aggarwal et al. [1] and Massa et al. [48]. This is for a number of reasons: First, from an economic standpoint, omitted variables, such as corporate culture, executive characteristics, and so on, are often difficult to quantify, and "heterogeneous" differences in companies, which are difficult to observe and measure, frequently lead to serious endogenous problems. The fixed-effect model can lessen the endogenous problem produced by the bias interference of omitted variables as the differential process in the estimated approach of the fixed-effect model can eliminate "time-invariant" firm heterogeneity to some extent. Second, this paper used statistical analysis such as the F test and the Hausman test from a statistical standpoint. The results reveal that at a 1% significance level, the fixed effect model is the most appropriate. The author may provide F test data upon request, and the values of Hausman test statistics can be found in Table 4. The Two-Stage Least Squares (2SLS) estimation results are used in this paper for the long-term holding type and the fast-in-fast-out type; simultaneously, the Limited Information Maximum Likelihood estimation (LIML) method, which is insensitive to weak instrumental variables, is used to obtain the model parameters. The reasonableness of the instrumental variable selection is indirectly validated if the difference between the two estimations is minimal. Thirdly, we diagnose the model residuals and draw a scatter plot of residuals and model fitting values at the same time, demonstrating that the model has no heteroscedasticity problem. Similarly, since the Generalized Method of Moments (GMM) regression method is suitable for the case where the model has heteroscedasticity, the GMM estimation method is not used in this paper. The regression results of the GMM method are available to the readers upon request. The regression findings are as follows: the value selection model results correspond to model (A), and hypothesis 1 is tested, as shown in Table 4. The value creation model's outcomes correlate to model (B), as shown in Table 5. Columns (3) and (4) of Table 5 tests hypothesis 2 and determines whether FIFO IIIs have a value creation function. The results in columns (1) and (2) test hypothesis 3 and determine whether long-term shareholding insurance institutions are value creators or value suppressors.”

At the same time, we attach the results of the regression of the GMM method here, but they are not used in the revised manuscript. The specific findings are listed in the table below:

**Table 4:** Value selection model (the explained variable is earnings per share:EPS)

|  | Long-term holding | | | Fast in and fast out | | |
| --- | --- | --- | --- | --- | --- | --- |
|  | IV（LIML） | IV（2SLS） | IV（GMM） | IV（LIML） | IV（2SLS） | IV（GMM） |
| VARIABLES | (1) | (2) | (3) | (4) | (5) | (6) |
|  |  |  |  |  |  |  |
| L. Shareh | **0.00463***** | **0.00463***** | **0.00456***** | **0.00509*** | **0.00509*** | **0.00537**** |
|  | **(0.001)** | **(0.001)** | **(0.001)** | **(0.003)** | **(0.003)** | **(0.002)** |
| L.EPS | 0.56547*** | 0.56546*** | 0.58517*** | 0.52198*** | 0.52199*** | 0.52754*** |
|  | (0.042) | (0.041) | (0.038) | (0.049) | (0.049) | (0.039) |
| Grow | 0.00068*** | 0.00068*** | 0.00068*** | 0.00065*** | 0.00065*** | 0.00064*** |
|  | (0.000) | (0.000) | (0.000) | (0.000) | (0.000) | (0.000) |
| AssLia | -0.00020 | -0.00020 | -0.00008 | -0.00057* | -0.00057* | -0.00050* |
|  | (0.000) | (0.000) | (0.000) | (0.000) | (0.000) | (0.000) |
| LogAss | -0.06924** | -0.06924** | -0.07287** | 0.02408 | 0.02408 | 0.02464 |
|  | (0.033) | (0.033) | (0.032) | (0.019) | (0.019) | (0.018) |
| Conc | 0.00117* | 0.00117* | 0.00113* | 0.00014 | 0.00014 | 0.00013 |
|  | (0.001) | (0.001) | (0.001) | (0.000) | (0.000) | (0.000) |
| NonIns | 0.00186*** | 0.00186*** | 0.00183*** | 0.00230*** | 0.00230*** | 0.00228*** |
|  | (0.000) | (0.000) | (0.000) | (0.000) | (0.000) | (0.000) |
| Industry variables | controlled | controlled | controlled | controlled | controlled | controlled |
| Joint significance | 40.96*** | 40.96*** | 43.45*** | 118.71*** | 118.72*** | 121.052*** |
| Under-identification | 1027.685*** | 1027.685*** | 1027.685*** | 175.280*** | 175.280*** | 175.280*** |
| Weak instrumental variables | 1681.579/ 199.382 | 1681.579/199.382 | 1681.579/199.382 | 3121.977/ 38.021 | 3121.977/ 38.021 | 3121.977/ 38.021 |
| Hansen J | 3.054 | 3.054 | 3.054 | 3.652 | 3.652 | 3.652 |
| Hausman |  | 199.49*** |  |  | 2459.68*** |  |
| Number of Observations N | 8715 | 8715 | 8,715 | 19500 | 19500 | 19500 |

**Table 5** Value creation model (the explained variable is the change in earnings per share, namely: D.EPS)

|  | Long-term holding | | | Fast in and fast out | | |
| --- | --- | --- | --- | --- | --- | --- |
|  | IV（LIML） | IV（2SLS） | IV（GMM） | IV（LIML） | IV（2SLS） | IV（GMM） |
| VARIABLES | (1) | (2) | (3) | (4) | (5) | (6) |
|  |  |  |  |  |  |  |
| D.Shareh | 0.0168** | 0.0167** | 0.0168** | -0.0081* | -0.0080* | -0.0068 |
|  | (0.008) | (0.008) | (0.008) | (0.005) | (0.005) | (0.004) |
| L2.EPS | -0.1920*** | -0.1919*** | -0.1889*** | -0.2310*** | -0.2310*** | -0.2277*** |
|  | (0.016) | (0.016) | (0.015) | (0.014) | (0.014) | (0.014) |
| Grow | 0.0006*** | 0.0006*** | 0.0006*** | 0.0005*** | 0.0005*** | 0.0005*** |
|  | (0.000) | (0.000) | (0.000) | (0.000) | (0.000) | (0.000) |
| AssLia | 0.0003 | 0.0003 | 0.0003 | -0.0003 | -0.0003 | -0.0003 |
|  | (0.000) | (0.000) | (0.000) | (0.000) | (0.000) | (0.000) |
| LogAss | -0.0659** | -0.0659** | -0.0619** | -0.0170 | -0.0170 | -0.0166 |
|  | (0.031) | (0.031) | (0.030) | (0.015) | (0.015) | (0.015) |
| Conc | -0.0004 | -0.0004 | -0.0002 | -0.0002 | -0.0002 | -0.0002 |
|  | (0.001) | (0.001) | (0.001) | (0.000) | (0.000) | (0.000) |
| NonIns | 0.0008** | 0.0008** | 0.0008** | 0.0008*** | 0.0008*** | 0.0008*** |
|  | (0.000) | (0.000) | (0.000) | (0.000) | (0.000) | (0.000) |
| Industry variables | controlled | controlled | controlled | controlled | controlled | controlled |
| Joint significance | 28.60*** | 28.61*** | 28.62*** | 49.38*** | 49.38*** | 50.15*** |
| Under-identification | 86.749*** | 86.749*** | 86.749*** | 219.641*** | 219.641*** | 219.641*** |
| Weak instrumental variables | 56.126 / 20.382 | 56.126 / 20.382 | 56.126 / 20.382 | 455.985 / 55.634 | 455.985 / 55.634 | 455.985 / 55.634 |
| Hansen J | 0.282 | 0.282 | 0.282 | 3.288 | 3.288 | 3.288 |
| Hausman |  | 693.99*** |  |  | 2548.53*** |  |
| Number of samples N | 8715 | 8715 | 8715 | 19500 | 19500 | 19500 |

***Reviewer’s Comment (Major Comment 4):***

*4. DATA PROCESSING AND DESCRIPTIVE STATISTICS*

***Reviewer’s Comment(Major Comment 4-(1)):***

*A better explanation of the three requirements you mentioned in line 388.*

**Authors’ Response (Major Comment 4-(1)):**

Thank you for your feedback on Line 388: " The selection of the data range is based on three different requirements". This original manuscript is not detailed enough to clarify the data range, resulting in confusion and difficulties in comprehending. As a result, the sample period has been restructured in this revised manuscript to make it more understandable. The following is an excerpt from the updated content (in blue):

Sample Period

The sample period for this study is from the first quarter of 2012 to the first quarter of 2018, and the research objects for empirical research are A-share listed companies on the Shanghai and Shenzhen stock markets in China. The data is collected on a quarterly basis, with a total of 25 quarters. Furthermore, while calculating the holding duration of IIIs, the first quarter of 2010 is used as the starting point, implying that the computation of the holding term of IIIs begins in the first quarter of 2010. Despite Chinese insurance funds having entered the capital market long before the subprime mortgage crisis, this paper uses 2010 as the starting point for the calculations because the Insurance Law empowering insurance companies to make equity investments was promulgated in 2009. According to statistics on publicly traded companies, insurance institutions owned less than 200 publicly traded companies in 2009, with a low shareholding ratio. Thus, this year is excluded and the calculations begin in 2010 as the effects of ownership was insignificant in 2009.

The data shows that as of the end of the first quarter of 2018, there were less than ten companies where insurance companies held shares for periods of more than 20 quarters (5 years). This indicates that our calculation of shareholding periods since 2010 essentially covers the shareholding cycle of all companies held by insured institutions and validates the starting point and the sample's integrity.

The period between 2010 and 2012 is not included in the sample as the holdings by insurance companies were not substantial during this period, and had a low shareholding ratio and a small scale [66]. Thus, the time period chosen is from the first quarter of 2012 up till the first quarter of 2018. China's capital market suffered stock market volatility in 2015. The "Vanke-Baoneng Battle" has raised concerns about insurance capital, as has the issue of employing short-term insurance funds for long-term investment by insurance capital. In July 2015, the Baoneng Group's Qianhai Life Insurance Company purchased Vanke shares on the secondary market, amounting for 5% of the company's total share capital. Baoneng, working with Qianhai Life Insurance, Jushenghua, and other parties through multi-party activities, exceeded China Resources Group, Vanke's original largest shareholder, for the first time in less than a month, and suggested calling an extraordinary general meeting to recall all directors. At the same time, Vanke used a private placement to introduce its shareholder Shenzhen Metro Group to pay the consideration. The probe has included the China Securities Regulatory Commission and the Shenzhen Stock Exchange. Shenzhen Metro Group eventually became the largest shareholder, Baoneng gradually withdrew from the competition, the former chairman of Vanke stepped down, and the war between Vanke and Baoneng came to an end. The sample in this research spans the three-year sample period before and after 2015, yielding a relatively complete capital market cycle. A balanced panel data sample with the data format "company-quarter" covering a total of 25 quarters for this study.

***Reviewer’s Comment(Major Comment 4-(2)):***

*Your sample started from 2012 to 2018 (25 quarters) but in line 411 you use data from 2010 to 2018 (33, segments). A better explanation is needed.*

**Authors’ Response (Major Comment 4-(2)):**

Thanks for the comment. As suggested, we've rewritten the "Sample Period" in the "Data" Section in order to distinguish between the sample period and the starting time of the insurance institution's shareholding computation.

Specifically, “The sample period for this study is from the first quarter of 2012 to the first quarter of 2018, and the research objects for empirical research are A-share listed companies on the Shanghai and Shenzhen stock markets in China. The data is collected on a quarterly basis, with a total of 25 quarters. Furthermore, while calculating the holding duration of IIIs, the first quarter of 2010 is used as the starting point, implying that the computation of the holding term of IIIs begins in the first quarter of 2010. Despite Chinese insurance funds having entered the capital market long before the subprime mortgage crisis, this paper uses 2010 as the starting point for the calculations because the Insurance Law empowering insurance companies to make equity investments was promulgated in 2009. According to statistics on publicly traded companies, insurance institutions owned less than 200 publicly traded companies in 2009, with a low shareholding ratio. Thus, this year is excluded, and the calculations begin in 2010 as the effects of ownership was insignificant in 2009.

The data shows that as of the end of the first quarter of 2018, there were less than ten companies where insurance companies held shares for periods of more than 20 quarters (5 years). This indicates that our calculation of shareholding periods since 2010 essentially covers the shareholding cycle of all companies held by insured institutions and validates the starting point and the sample's integrity.

The period between 2010 and 2011 is not included in the sample as the holdings by insurance companies were not substantial during this period and had a low shareholding ratio and a small scale [66]. Thus, the time period chosen is from the first quarter of 2012 up till the first quarter of 2018. China's capital market suffered stock market volatility in 2015. The "Vanke-Baoneng Battle" has raised concerns about insurance capital, as has the issue of employing short-term insurance funds for long-term investment by insurance capital. In July 2015, the Baoneng Group's Qianhai Life Insurance Company purchased Vanke shares on the secondary market, amounting for 5% of the company's total share capital. Baoneng, working with Qianhai Life Insurance, Jushenghua, and other parties through multi-party activities, exceeded China Resources Group, Vanke's original largest shareholder, for the first time in less than a month, and suggested calling an extraordinary general meeting to recall all directors. At the same time, Vanke used a private placement to introduce its shareholder Shenzhen Metro Group to pay the consideration. The probe has included the China Securities Regulatory Commission and the Shenzhen Stock Exchange. Shenzhen Metro Group eventually became the largest shareholder, Baoneng gradually withdrew from the competition, the former chairman of Vanke stepped down, and the war between Vanke and Baoneng came to an end. The sample in this research spans the three-year sample period before and after 2015, yielding a relatively complete capital market cycle. A balanced panel data sample with the data format "company-quarter" covering a total of 25 quarters for this study.”

***Reviewer’s Comment(Major Comment 4-(3)):***

*It is important to specify whether you are using a balanced or unbalanced panel. I think, reading your data in the regression tables that it is balanced, but you need you said clearer. But, if so, if it is balanced, you need to take care of the so-called “survival bias” effect in panel data set.*

**Authors’ Response (Major Comment 4-(3)):**

Thank you for your suggestion. As you mentioned, our data is balanced. In the “Data” section of revised manuscript, we have meticulously detailed the data structure. The following is an excerpt of the specified content (marked in blue in “Data” section and “Selection of Instrumental variables” section):

“......A balanced panel data sample with the data format "company-quarter" covering a total of 25 quarters for this study.”

“The data in this research is balanced panel data, and we use the panel data to develop a fixed effect model. The fixed effect model can ignore the influencing factors of individuals that do not change with time such as corporate culture, company creation date, corporate character, and other factors...”

We have carefully considered the possible "survival bias" mentioned by the reviewer. First, this paper excludes from the sample the "ST" companies who have been warned by regulatory agencies, implying that the value selection model may have "survival bias." However, the value creation model's construction mitigates this issue to some extent. The dependent variable we choose in the value creation model is the change of the company's value rather than a static indicator, so that we can see how the company's value changes are affected by the insurance institution's shareholding. And we use the instrumental variable approach to address endogeneity as much as possible. As a result, the value creation model presented in this paper is better suited to avoiding the survival bias. Second, in the counterfactual test model, if the survival bias is large, the coefficients before the shareholding ratio of insurance institutions in models (C) and (D) should also have a positive relationship with the company value in the previous period, but the coefficient before the shareholding ratio of insurance institutions in model (C) and (D) (see Table 6) does not show significance, indicating that the influence of survival bias in this paper is relatively small to some extent. Finally, this research uses the same sample selection procedure following Aggrawal et al. (2011) and Cornett et al. (2007) on the impact of institutional investors on corporate performance. Therefore, the survival bias has a minor impact.

***Reviewer’s Comment(Major Comment 4-(4)):***

*In line 407, you use the terms “ST” and “*ST”, again two new concepts suddenly shown in the paper. What do they stand for?*

**Authors’ Response (Major Comment 4-(4)):**

Thanks for the reminder. Regarding the issue of the concept not being well explained, the new version of this article has been checked and improved. The following is an excerpt of the specified content (marked in blue in “Data” section):

“This paper excludes abnormal samples such as "ST" and "*ST" shares, as well as the sample of financial companies, which yielded 1,654 samples. If a listed company's financial state or other conditions are anomalous in China's capital market, the Shanghai and Shenzhen Stock Exchanges will give the listed company's shares special treatment. This form of stock is known as ST stock because of the "special treatment" it receives. If a stock's name is prefixed with ST, it carries investment risk; if *ST is added, it has the danger of being delisted.”

***Reviewer’s Comment(Major Comment 4-(5)):***

*From line 418 to 416, what is it the “conversion” between x-times (e.g. 1.4 times) and quarters? Besides, why you select the criterium of “more than 11 quarters and less than 4”? Please, explain and support with the literature.*

**Authors’ Response (Major Comment 4-(5)):**

Thanks for the comment. We rearranged this piece of the writing in amended content to make the presentation clearer, based on the reviewers' questions. The following is an excerpt from the revised manuscript (last paragraph on p. 26):

“The average holding time of the selected sample by insurance firms is 6.9 quarters, according to the aforementioned method. Elyasiani and Jia [24] divided the long-term and FIFO samples based on whether it was greater than the index mean. This work addresses the symmetry of the long and short multiples to assure the sample's adequacy while also distinguishing the structural characteristics of the holding duration. IIIs with more than 1.25 times the average holding time (greater than 9 quarters) are considered long-term holding types in this paper, while those with less than 0.75 times (less than 6 quarters) are considered FIFO holding types. The ratio is set at 1.5 times and 0.5 times, respectively, in the robustness test, i.e., more than 11 quarters are recorded as longer-term holdings, and less than 4 quarters are recorded as shorter-term holdings.”

Here is the table describing our calculations.

|  |  | Actual coefficient | Rounding | Final Choice |
| --- | --- | --- | --- | --- |
| Average | 6.9 | 6.9 | 6.9 | 6.9 |
| Long Term | 6.9*1.25 | 8.625 | 9 | >=10 |
| Short Term | 6.9*0.75 | 5.175 | 6 | <=5 |
| Longer Term | 6.9*1.5 | 10.35 | 11 | >=12 |
| Shorter Term | 6.9*0.5 | 3.45 | 4 | <=3 |

***Reviewer’s Comment(Major Comment 4-(6)):***

*You must improve your description of the control variables. You does not describe all of them. What is “agency variable”? (again, suddenly, you use a new concept).*

**Authors’ Response (Major Comment 4-(6)):**

Thank you for highlighting the flaws in our paper. All variables are defined in greater detail in the revised version, and citations have been provided. After reviewing various literature, we determined that the expression "agency variable" was ambiguous, thus it was modified to "alternative proxies". This study uses three indicators to measure company value, with EPS earnings per share serving as the primary measuring indicator and ROA and ROE serving as alternative proxies for the robustness test. Other control variable descriptions have also been improved; see the above Authors' Response (Major Comment 3-(3)). The following is an excerpt from the revised manuscript (marked in blue on p. 18):

“This paper determines the indicators to measure the company's value after establishing the models (A) and (B). Most studies, like those by Cornett et al. [18], Ferreira and Matos [26] and Healy [32], employ Tobin's Q [33] value to evaluate firm value, but China's stock market's price discovery function is in a nascent stage, and there is a strong speculative atmosphere. It is difficult to determine the true worth of publicly traded companies based on Tobin’s Q [33]. Therefore, this article selects three measures to estimate the value of organizations after meticulous screening. Our first indicator, based on Chaganti and Damanpour [11] and Tang and Song [66], chooses earnings per share (EPS) as the key explanatory variable. Second, two alternative proxies, return on assets (ROA) [18, 36] and return on equity (ROE) [41, 63], are utilized as robustness test indicators...”

***Reviewer’s Comment(Major Comment 4-(7)):***

*In Table 2, have you checked whether companies with many negative values in the dependent variables behave differently than companies with positive? Could it be possible to show a different behaviour? How have you treated outliers?*

**Authors’ Response (Major Comment 4-(7)):**

Thanks for the comment. According to the reviewer's suggestion, we have organized the data of this article, and selected companies with negative company values (EPS, ROA, ROE) for descriptive statistics to observe their behaviors.

First and foremost, the numerator of the company value variable used in this research is based on net profit, which can be positive or negative, therefore the three indicators will find that the company value is negative in descriptive statistics.

Then, we selected the indicators with negative EPS, ROA, and ROE values and examined the shareholding ratio and shareholding period of insurance institutions, as shown in the table below:

|  | EPS<0 | ROA<0 | ROE<0 | Full Sample |
| --- | --- | --- | --- | --- |
|  | Mean | Mean | Mean | mean |
| Shareholding ratio of insurance institutions | 0.5392 | 0. 5198 | 0.5344 | 0.6921 |
| Insurance institution shareholding period | 0.9403 | 0. 9204 | 0.9518 | 1.3488 |

The data shows that the shareholding ratio and shareholding period of insurance institutions are lower than the average in companies with negative company values. We changed the descriptive statistics section to better illustrate this comparison in the revised manuscript, including a comparison of shareholding ratio and holding period following the company's value grouping and describing the circumstances of outlier processing. Details can be found in the revised manuscript, and the following is an excerpt:

“All variables are winsorized by 1 percent and 99 percent in this work to eliminate the influence of outliers on the model. Table 2 presents summary statistics for the variables. Panel A shows that long-term and FIFO III shareholding ratios are roughly 1.7417% and 0.2414%, respectively, with long-term investors having the greatest shareholding ratio of 58.820%. FIFO investors had the highest shareholding ratio at 21.820%.The ratio of the total shareholding ratio of insurance institutional investors to the total number of quarters is used to calculate the average shareholding ratio of insurance institutional investors. If the quarters with a zero-shareholding percentage are omitted, the overall sample's average shareholding ratio is around 2.5 percent. In addition, Panel B shows the shareholding ratio and shareholding term of insurance institutions under various company values after grouping company value EPS according to the average value. The mean shareholding ratio and holding term of insurance institutions are significantly higher in the group with higher company value (EPS>=median) than in the group with lower business value (EPS<median). It is first demonstrated that IIIs have a value selection function."

**Table 2:** Descriptive Statistical Analysis

| Pan Long-term holding | | | | | | | | | | | | | | | |
| --- | --- | --- | --- | --- | --- | --- | --- | --- | --- | --- | --- | --- | --- | --- | --- |
| Index | EPS | *ROA* | | *ROE* | Shareh | | Insfir3 | Insfir5 | Grow | | AssLia | *Size* | | Conc | NonIns |
| Number of samples | 8700 | 8700 | | 8700 | 8700 | | 8700 | 8700 | 8700 | | 8700 | 8700 | | 8700 | 8700 |
| Mean | 0.4671 | 4.6927 | | 5.3377 | 1.7417 | | 0.8857 | 1.3346 | 15.8804 | | 47.4281 | 2.0051 | | 49.8864 | 6.2176 |
| Standard deviation | 0.5216 | 5.3864 | | 5.8877 | 4.077 | | 3.2786 | 3.7656 | 96.4969 | | 19.5290 | 0.6158 | | 16.6336 | 7.8271 |
| Minimum | -0.8344 | -29.4725 | | -10.54 | 0 | | 0 | 0 | -235.300 | | 7.7649 | 0.2612 | | 8.4986 | 0 |
| Median | 0.3553 | 3.8896 | | 4.08 | 0.33 | | 0 | 0 | 7.5449 | | 47.0514 | 1.9206 | | 50.2304 | 3.585 |
| Maximum | 2.2213 | 46.3117 | | 21.4900 | 58.82 | | 36.3737 | 46.8682 | 324.8260 | | 86.358 | 4.3885 | | 98.3571 | 102.762 |
| Fast in and fast out | | | | | | | | | | | | | | | |
| Number of samples | 19500 | 19500 | | 19500 | 19500 | | 19500 | 19500 | 19500 | | 19500 | 19500 | | 19500 | 19500 |
| Mean | 0.2851 | 3.7367 | | 4.1145 | 0.2414 | | 0.0617 | 0.1215 | 18.1226 | | 42.9522 | 1.6296 | | 47.5812 | 4.9567 |
| Standard deviation | 0.4033 | 5.8575 | | 5.784 | 0.8611 | | 0.5594 | 0.6989 | 114.5397 | | 21.5781 | 0.5117 | | 15.18 | 7.7091 |
| Minimum | -0.8344 | -29.4725 | | -10.54 | 0 | | 0 | 0 | -235.294 | | 3.8217 | -0.598 | | 0.5648 | 0 |
| Median | 0.2157 | 3.2157 | | 3.05 | 0 | | 0 | 0 | 5.1728 | | 41.8841 | 1.5647 | | 46.9198 | 1.9931 |
| Maximum | 2.2213 | 46.3117 | | 21.49 | 21.82 | | 18.75 | 18.75 | 324.826 | | 92.1799 | 3.4195 | | 97.53 | 96.6152 |
| Panel B | | |  | | | EPS>median | | | | EPS<median | | | Difference | | |
| Long-term holding | | | | | | | | | | | | | | | |
|  | | |  | | | Mean | | | | Mean | | | Mean | | |
| Shareholding ratio | | | | 2.0480 | | | | 1.4864 | | | | 0.5616*** | | | |
| Holding period | | | | 4.5037 | | | | 3.1912 | | | | 1.3125*** | | | |
| Fast in and fast out | | | | | | | | | | | | | | | |
| Shareholding ratio | | | | 0. 2792 | | | | 0. 1858 | | | | 0.0934*** | | | |
| Holding period | | | | 0.3247 | | | | 0.2364 | | | | 0.0883*** | | | |

***Reviewer’s Comment (Major Comment 5):***

*5. MODEL REGRESSION ANALYSIS AND CONTERFACTUAL TEST*

***Reviewer’s Comment (Major Comment 5-(1)):***

*Table 4 and 5 do not show the same format.*

*At the bottom of the regression tables, I suggest using the name of the tests applied (Hansen, Sargan), it could help.*

**Authors’ Response (Major Comment 5-(1)):**

Thank you for your reminding and suggestions. According to your comments, the revised manuscript has revised all of the tables in the empirical section, altered the decimal places in all of the tables to ensure consistency, and retained four digits after the decimal point equally.

Because the LIML and 2SLS findings are so close in Table 4, the difference starts at the fifth digit after the decimal point, hence Table 4 keeps five decimal places. In the revised version, we've provided a description. The following is an excerpt:

“The numbers in this paper's table are rounded to four decimal places, however Table 4 is rounded to five decimal places because the LIML and 2SLS findings only differ in the fifth decimal place...”

At the same time, referring to other literatures, the meaning of each statistic in the table is marked in detail at the bottom of each table, and the Hansen J statistic report is added. For more information, see Tables 4 and 5 in the revised manuscript.

***Reviewer’s Comment (Major Comment 5-(2)):***

*In Table 6, are you using LIML or 2SLS?*

*Is there a connection between Table 6 and the hypotheses?*

**Authors’ Response (Major Comment 5-(2)):**

Thanks for your comment. Tables 6, 7, and 8 all use LIML, however we didn't describe it in the original manuscript, as the reviewer pointed out. The description of the regression methods in Tables 6 to 8 is added to the revised version, and it is also included in the table annotations. The following is an excerpt on page 37 from the revised manuscript:

“The regression findings of the LIML and 2SLS approaches do not differ significantly, as shown in tables 4 and 5. Thus, we only employ the LIML approach for regression in the following models...”

Secondly, Table 6 shows the counterfactual test findings, which correspond to the counterfactual test models (C) and (D) built in "Model Design". The counterfactual test model is used in this research to see if the endogeneity problem is effectively handled in model (A) and (B) after applying the instrumental variable method and the explanatory variable lag approach. The following is our new expression:

“Theoretically, IIIs' future shareholding should have no impact on the existing holding company's performance. When the parameters $\beta_{j1}$ and $\gamma_{j1}$ to be estimated are significant in the measurement model constructed by the future shareholding ratio, the information about the insurance company’s equity structure indicators and the held company’s performance is still in the model’s disturbance terms, and as an instrumental variable, it does not effectively solve the model’s endogeneity. Similarly, when they are not significant, the endogenous problem in the model is effectively controlled to some extent...” (last paragraph on p. 21)

“We built models (C) and (D) for counterfactual testing in the preceding discussion. Theoretically, future III shareholding will have no impact on the current holding company's value, i.e., the shareholding ratio of insurance institutions in period t+1 has no impact on the firm's worth in period t...” (first paragraph on p. 36)

***Reviewer’s Comment (Major Comment 6):***

*6. FURTHER STUDY*

***Reviewer’s Comment (Major Comment 6-(1)):***

*Why is the support (citations) used in the new “classification criteria” shown in this section?*

**Authors’ Response (Major Comment 6-(1)):**

Thanks for the comment. This paper, after amendment, discusses this classification standard in greater detail, and the following is an excerpt of the content:

“The sample type - categorization criteria, is further narrowed in this research to explore the performance of value selection and creation methods across longer and shorter holding periods. This classification is a more in-depth research challenge and a strong evaluation of the results of this work from a different perspective. The comparison also reflects the stability of the value creation and value selection functions...”(First paragraph in the “Further Research” Section)

***Reviewer’s Comment (Major Comment 6-(2)):***

*In line 549, I might be wrong, but I think that the significance level does not improve as you stated.*

**Authors’ Response (Major Comment 6-(2)):**

Thanks for your valuable comments. Following the change, this article has included a more extensive discussion of the comparison between the results in Table 8 and the results in the preceding table, with the comparison of coefficients and significance level highlighted to make the comparison clearer and boost readability. The following content is added in the first paragraph of the “Further Research” Section:

“The average holding period of insurance institutions above 1.5 times is recorded as a sub-sample with a longer holding period (long-term holding type 2). Similarly, holding periods of less than 0.5 times are recorded as a subsample of FIFO holdings with more frequent investment conversions (FIFO type 2), and model parameters are estimated using the value-selection model (A) and value-selection model (B). Table 8 displays the model's output. The FIFO type 2 IIIs have a more obvious value selection function than the FIFO type IIIs in table 4, as shown in table 8. The coefficient increased from 0.00509 (column (3) of table 4) to 0.0170 (column (2) of table 8), and the significance level increased from 10% to 5%, indicating that IIIs with shorter-term investment horizons face greater pressure to maintain the value of debt funds, and will actively utilize its advantages in information collection to seek high-quality companies to maximize the value of their debt funds.

Long-term holding type 2 has a substantial value selection function with a slight improvement in the coefficient (0.0048 (column (1) of Table 8) > 0.00463 (column (1) of Table 4), indicating that insurers are more cautious for longer-term holdings. The value creation effect of long-term holding type 2 IIIs is stronger in the value creation model, with the coefficient increasing from 0.0168 (column (1) of table 5) to 0.0245 (column (3) of table 8). Despite the decrease in significance level, it illustrates that the more IIIs whose purpose is to hold shares for a long time, the more motivated they are to participate in corporate supervision and corporate governance, resulting in a value-creating influence on the company they own. The FIFO type 2 still lacks a value creation function and has a detrimental impact. ”

***Reviewer’s Comment (Major Comment 6-(3)):***

*In line 557 I believe that “0.00463” is wrong, and the right number is “0.017”. In any case, explain better which tables you are comparing to avoid any misunderstanding.*

**Authors’ Response (Major Comment 6-(3)):**

Thanks for your comment. In the revised manuscript, the numbers in Tables 4 and 5 in the comparison of line 557 have been revised, and the associated tables and columns have been marked in detail following the numbers to avoid misunderstanding. Also see Authors' Response (Major Comment 6-(2)).

***Reviewer’s Comment (Major Comment 6-(4)):***

*In lines 563-564, what does it add this new “perspective”? Why?*

**Authors’ Response (Major Comment 6-(4)):**

Thanks for the comment. In further research, this paper makes the categorization standard tighter. In particular, “The sample type - categorization criteria, is further narrowed in this research to explore the performance of value selection and creation methods across longer and shorter holding periods. This classification is a more in-depth research challenge and a strong evaluation of the results of this work from a different perspective. The comparison also reflects the stability of the value creation and value selection functions.” Also see the first paragraph in the “Further Research” Section. This new perspective refers to extend long-term period and shorten the short-term period. In this way, it is once again demonstrated that empirical results are robust.

***Reviewer’s Comment (Major Comment 6-(5)):***

*Again, in Table 7, (Why 7.1?), are you using LIML or 2SLS?*

**Authors’ Response (Major Comment 6-(5)):**

Thank you for pointing up the inconsistencies in this article. The headers of the revised version have been meticulously numbered and checked for consistency and accuracy. The regression methods that were utilised in each table are also described in the table's notes. The LIML regression approach is used in the amended Table 8 (that is, Table 7 in the original manuscript). We also explain why we utilize the LIML approach in the text "counterfactual testing," and the following material is copied from the first paragraph of the "Counterfactual Test" section:

“The regression findings of the LIML and 2SLS approaches do not differ significantly, as shown in tables 4 and 5. Thus, we only employ the LIML approach for regression in the following models...”
