# Supplementary material for: The impact of insurance institutional investors on corporate value from selection and creation perspective
Source: PLoS One. 2022 Jul 1;17(7):e0269520. doi: 10.1371/journal.pone.0269520 (PMC9249200; doi:10.1371/journal.pone.0269520)
Supplement: Attachment — Submitted filename: Responses to Reviewer 2022_05_15.docx [file pone.0269520.s003.docx]

# Responses to Reviewer1:

Our sincere thank you to the reviewer for the very constructive comments on our manuscript. Your constructive comments on the paper were extremely valuable in improving the quality of the paper. In what follows, your comments are shown in italics and followed by our point-by-point responses. All the page numbers refer to those in the revision. We believe that you will find this report and our revised manuscript have satisfactorily addressed the issues you have raised, and we thank you in advance for your valuable time in reviewing the manuscript.

***Reviewer’s Comment:***

*1. The paper would benefit (in my view) if you put the Sample Description section first, and then all the content related to Model Design and Variables.*

**Authors’ Response:**

Much appreciated for the suggestions. We put Model Design and Variables upfront because instrumental variables and model design are closely related. Descriptions of all samples may be added in Sample Description Section. Had we put the Sample Description section first, it would have been the situation where the instrumental variables were described before we justified the reasons for selecting those variables. Furthermore, we would need to change quite a few logics if we were to make some adjustments. And we also reviewed the article published on Plos One. There is not a standard answer to whether introducing the data first or introducing the model first. For example, Jiang et al. (2019)^[1]^ first described the model and then the variables, and finally the samples in “The impact of the inclusive financial development index on farmer entrepreneurship” on Plos One. We accepted all your previous suggestions but this one. We sincerely appreciate your understanding and thank you for keeping up with us and helping us improve all this time.

***Reviewer’s Comment:***

*2.References should be ordered throughout the article according to PLOS ONE guidelines. That is, the first reference in the paper should be number 1, the next number 2, and so on.*

**Authors’ Response:**

We appreciate the suggestions by the reviewer. We were being careless about it. Changes were made in the revised manuscript.

# Responses to Reviewer2:

Our sincere thank you to the reviewer for the very constructive comments on our manuscript. Your constructive comments on the paper were extremely valuable in improving the quality of the paper. In what follows, your comments are shown in italics and followed by our point-by-point responses. All the page numbers refer to those in the revision. We believe that you will find this report and our revised manuscript have satisfactorily addressed the issues you have raised, and we thank you in advance for your valuable time in reviewing the manuscript.

***1. ABSTRACT***

***Reviewer’s Comment (Major Comment 1):***

*I recommend not to be so categorical when you state that “this study rectifies”. I could be much better to use a softer or smoother expression, such as “shed light”, “provides more insight…”.*

**Authors’ Response :**

We appreciate the suggestions by the reviewer. Changes were made in the revised manuscript.

***2. INTRODUCTION***

***Reviewer’s Comment (Major Comment 2-（1）):***

*Although in the Abstract you explain what a IIIs is, I think that it is necessary to explain it again in this section.*

**Authors’ Response (Major Comment 2-（1）):**

We appreciate the suggestions by the reviewer. We have added it in the introduction.

***Reviewer’s Comment (Major Comment 2-（2）) :***

*When you start describing the three roles (line 53) of institutional investors, I think that you need to help readers to distinguish these three roles in the following paragraphs. You me it took time to distinguish them.*

**Authors’ Response (Major Comment 2-（2）) :**

We appreciate the suggestions by the reviewer. We added (1) (2) (3) to distinguish these three roles on page 4. It certainly becomes clearer after we fixed it.

***Reviewer’s Comment (Major Comment 2-（3）) :***

*In lines 136-138 you repeat the “Vanke-Baoneng Battle” issue as you did in lines 45-47 using almost the same expression.*

**Authors’ Response (Major Comment 2-（3）) :**

We appreciated the suggestions by the reviewer. We have made line 136 through line 138 more concise to avoid unnecessary repetitiveness. Now Line 137 through line 139 is as follows: “**This article disproves the notion that insurance funds are "barbaric funds" and provides factual support for Chinese regulators' long-term investment strategy for insurance businesses.”**

***3. LITERATURE REVIEW / THEORETICAL ANALYSIS / HYPOTHESES***

***Reviewer’s Comment (Major Comment 3):***

*From my point of view a brief definition of the two types of investment roles would be welcome: value selection and value creation.*

**Authors’ Response ：**

We appreciated the suggestions by the reviewer. We have already added a brief definition of value selection in line 151 to line 153, “This paper defines value selection as the insurance companies are able to select companies with development potential that will grow in value in the future to benefit shareholders”, and value creation in line 187 to line 188, “Value creation is defined as the value of the held company will increase in value because of insurance company’s holding after the insurance company holds shares or increases the original holdings”.

***Reviewer’s Comment(Major Comment 3-(1)):***

*Regarding the three hypotheses, and to make you think for future papers, it would be interesting to analyse whether these hypotheses varies as the percentage of shares (Shareh variable) also varies, using for example, quartiles.*

**Authors’ Response (Major Comment 3-(1)):**

We appreciated the suggestions by the reviewer. As the reviewer said, it is more interesting to observe the performance of value selection and value creation under different shareholding ratios of insurance institutions. Following your suggestions, we try to use the method of quantile grouping to test the function of value selection and value creation, and find that the hypothesis of this paper mainly exists in the group with higher shareholding ratio of insurance institutions. Due to the limitation of space and structure, this paper does not supplement this content. In the subsequent research on the shareholding of insurance institutions, we will carefully assess the impact of the difference in the shareholding ratio of insurance companies. We have also added your suggestions to the section in future research directions, thank you for always helping us improve all this time.

***Reviewer’s Comment(Major Comment 3-(2)):***

*What is the added value of H2 compared to the empirical evidence of references 10, 44, 72 and 73? If this papers confirm that short-term IIIs’ stock investments cannon improve company performance, what are you added to the literature? Explain a little bit.*

**Authors’ Response (Major Comment 3-(2)):**

We thank the reviewer for the questions. Institutional investors studied in references 37, 38, 39 and 40 included funds, securities companies, and financing guarantee companies, but did not involve insurance institutional investors at all. The added value in this paper is reflected in a separate study of insurance institutional investors. This is rarely mentioned in the previous literature and broadens the scope of the study.

***4. MODEL DESIGN AND VARIABLES***

***Reviewer’s Comment(Major Comment 4-(1)):***

*Citations from the literature justifying or supporting the “Model design for counterfactual testing” would be also welcome.*

**Authors’ Response (Major Comment 4-(1)):**

We appreciated the suggestions by the reviewer. Based on the reviewer’s suggestion, we added the classic literature referenced by this method in Counterfactual Test section. The modified excerpts are as follows: “**We built models (C) and (D) for counterfactual testing in the preceding discussion (Fiona et al., 1999; Fernandez, 2014).**”

***Reviewer’s Comment (Major Comment 4-(2)):***

*In your answers you mention that your paper follow Arellano and Bond (1991) but I cannot see the citation in the paper.*

**Authors’ Response (Major Comment 4-(2)):**

We appreciated the suggestions by the reviewer. We quoted it in line 394 and we are really sorry about it. It might be because our citation is not standard so that you missed it. Therefore references have been ordered throughout the article according to PLOS ONE guidelines.

***5. DATA PROCESSING AND DESCRIPTIVE STATISTICS***

***Reviewer’s Comment (Major Comment 5-(1)):***

*Again, to improve the paper or for a new one, I think that you could test whether your results hold up or change for only observations with positive EPS, ROA or ROE. Sometimes when companies are going through serious difficulties their normal “roles or priorities” change.*

**Authors’ Response (Major Comment 5-(1)):**

Many thanks to the reviewers for your ideas on the future research directions of our paper. This paper takes into account the loss in the process of enterprise operation in the sample, because it is considered that enterprise profit and short-term loss are a normal state of enterprise operation. The samples with EPS<0 only account for 7.8% of the total samples in this paper, which is relatively small and the sample size is also small, which makes the significance of its separate research relatively limited. In addition, due to the space and structure limitation of this paper, the samples with EPS<0 were not tested separately.

But as the reviewer said, when companies are going through serious difficulties their normal “roles or priorities” may change as well. As for future research, we will focus on assessing the differences in the roles of companies in the state of profit and loss. We have also added your suggestions to the future research directions section, thank you for always helping us improve all this time.

***Reviewer’s Comment (Major Comment 5-(2)):***

In Table 3, coefficient between Grown and Conc, there are five digits after dot (0.00300).

**Authors’ Response (Major Comment *5-(2)*):**

We appreciated the suggestion by the reviewer. It’s our fault for being careless. The last two decimals have been deleted.

***6. MODEL REGRESSION ANALYSIS AND CONTERFACTUAL TEST***

***Reviewer’s Comment (Major Comment 6-(1)):***

In Table 5 you forgot to add the “Note” statements.

**Authors’ Response (Major Comment 6-(1)):**

We appreciated the suggestion by the reviewer. Because the “Note” statements of Table 4 and Table 5 are the same, we wrote “this also applies to Table 5” below Table 4. However, based on the reviewer’s suggestion, we have once again added the “Note” statements in Table 5.

1. Jiang L, Tong A, Hu Z, Wang Y (2019). The impact of the inclusive financial development index on farmer entrepreneurship. PLoS ONE 14(5): e0216466. https://doi.org/10.1371/journal. pone.0216466
